# Application of *Saccharomyces cerevisiae*/Calcium Alginate Composite Beads for Cephalexin Antibiotic Biosorption from Aqueous Solutions

**DOI:** 10.3390/ma14164728

**Published:** 2021-08-21

**Authors:** Lăcrămioara Rusu, Cristina-Gabriela Grigoraș, Andrei-Ionuț Simion, Elena Mirela Suceveanu, Daniela Șuteu, Maria Harja

**Affiliations:** 1 Department of Chemical and Food Engineering, “Vasile Alecsandri” University of Bacău, 600115 Bacău, Romania; lacraistrati04@yahoo.com (L.R.); asimion@ub.ro (A.-I.S.); mirela.suceveanu@ub.ro (E.M.S.); 2 Department of Organic, Biochemical and Food Engineering, “Gheorghe Asachi” Technical University of Iași, 700050 Iași, Romania; danasuteu67@yahoo.com; 3 Department of Chemical Engineering, “Gheorghe Asachi” Technical University of Iași, 700050 Iași, Romania; maria_harja06@yahoo.com

**Keywords:** antibiotic, cephalexin, biosorption, *Saccharomyces cerevisiae*, calcium alginate, water treatment, kinetics models, adsorption isotherms

## Abstract

Cephalexin (CPX) is recognized as a water pollutant, and it has been listed in a number of countries with a risk factor greater than one. Herein, the present work focused on the synthesis, characterization and biosorption capacity evaluation of *Saccharomyces cerevisiae* immobilized in calcium alginate as a biosorbent to remove CPX from aqueous solutions. Biosorbent was characterized by SEM and FTIR techniques. Batch biosorption experiments were conducted in order to evaluate the effect of the initial pH, biosorbent dose and CPX initial concentration. The removal efficiency, in considered optimal conditions (pH = 4, CPX initial concentration = 30 mg/L, biosorbent dose = 1 g/L) was 86.23%. CPX biosorption was found to follow the pseudo–second-order kinetics. The equilibrium biosorption data were a good fit for the Langmuir model with correlation coefficient of 0.9814 and maximum biosorption capacity was 94.34 mg/g. This study showed that the synthesized biosorbent by immobilization technique is a low-cost one, easy to obtain and handle, eco-friendly, with high feasibility to remove CPX antibiotic from aqueous solution. The findings of this study indicate that the biosorbents based on microorganisms immobilized on natural polymers have the potential to be applied in the treatment of wastewater.

## 1. Introduction

Pollution of the environment by pharmaceuticals is a challenging issue [1,2,3,4,5]. In fact, the development of analytical techniques has allowed their quantification in different environmental matrices [6,7]. 

Numerous studies have reported that organic pollutants such as drugs can have adverse effects on various life forms [2,3]. 

These pollutants have a potential environmental risk, especially to water quality, and they also generate a number of toxic effects because these molecules are biologically active [1,8,9,10].

Antibiotics are a category of drugs consumed in large quantities worldwide, so they are found in different water categories (wastewater, surface water and groundwater) [2,11]. 

Drugs in general, and antibiotics in particular, cannot be removed in wastewater treatment plants, due to the fact that the involved processes are generally based on physical and biological processes. 

This fact leads to the drugs’ presence in the effluents of the wastewater treatment plants [2,12], so implicitly to the contamination of water resources, which involves serious problems for the environment and human health, such as toxicity and/or generation of antibiotic-resistant bacteria [13].

Cephalexin (CPX) (C_16_H_17_O_4_ N_3_S·H_2_O) is a first-generation cephalosporin antibiotic with molecular weight of 365.40 g/mol, that was approved by FDA in 1971. It is used for the treatment of diseases infection caused by bacteria such as *Streptococcus pneumoniae*, *Staphylococcus aureus*, *Escherichia coli* etc. [14,15].

Due to its wide spectrum of action, the consumption of cephalexin in the world is estimated at around 3000 t/year [16]. 

Cephalexin has been detected in wastewater, sewage effluents, hospital effluents, surface water, in various concentrations between 5000 ng/L and 7.4 ng/L [17,18,19,20].

CPX has been listed in a number of countries with a risk factor greater than one due to its high potential to induce antimicrobial resistance in bacterial cells, leading to mutagenic and carcinogenic effects [21,22].

Additionally, a series of studies correlate the use of CPX-contaminated water with a number of symptoms such as diarrhea, skin irritation, nausea and stomach ache [23].

Thus, this antibiotic must be effectively removed before being discharged into the environment to avoid its negative impact.

The literature presents numerous technologies for CPX removal, which include adsorption, photocatalysis, electrocoagulation, nanotechnology, etc. [14].

Advanced oxidation processes have been used successfully for CPX removal, but these processes have a number of disadvantages, such as the generation of more toxic degradation products and high operating costs [24,25,26,27].

Among these processes, adsorption is an interesting option for CPX removal due to the fact that this process is simple, efficient and economical.

The numerous studies regarding the removal of CPX from aqueous solutions with different types of adsorbents have been mentioned in the literature in the last decade. Among these, we can cite: 

Activated carbon obtained from different materials: biochar obtained from oil palm fiber [2]; original and Cu(II)/Fe(III) impregnated activated carbons developed from lotus stalks [28]; activated carbon nanoparticles prepared from vine wood [29]; activated carbon prepared from alligator weed [30]; walnut shell-based activated carbon [16]; *Anthriscus sylvestris*-derived activated biochar [31]; activated carbon (PPAC) derived from pomegranate peel [32]; activated carbons from *Albizia lebbeck* seed pods by microwave-induced KOH and K_2_CO_3_ activations [33].

Nanomaterials: PPAC-ZnO and PPAC-nZVI nanocomposite using pomegranate peel [34]; boron nitride nanosheets [35]; Cu–Zn bionanocomposite biosynthesized in secondary metabolic products of *Aspergillus arenarioides* EAN603 with pumpkin peels medium [36], and modified biochar supported Ag/Fe nanoparticles [37]. 

Zeolites: zeolite coating with magnetic Fe_3_O_4_ nanoparticles [38]; natural zeolite and zeolite coated with manganese oxide nanoparticles [39], and magnetic zeolite [40].

Other materials: octenyl succinic anhydride starch [1]; zirconium-based metal-organic framework [41]; phosphoric acid-activated chitin [23]; magnetic carbon obtained from poly (ethylene) and terephthalate (PET) wastes [42].

Studies on CPX biosorption on living/dead microorganisms/biomass are also presented, among which are bacterial consortium biomass (living and dead) [43]; non-living *Chlorella* sp., biomass [44]; mixed bacterial cell biomass (living cells) [45] and *Bacillus subtilis* living cells [46].

The main problem that arises when using microorganisms or biomass is the separation of supernatants, which leads to the conclusion that it is necessary to obtain biosorbents that are easy to handle and separate and at the same time environmentally friendly.

Taking into account those mentioned above, immobilization or encapsulation of microorganisms or biomass on inert supports would be a viable solution. This fact was demonstrated in our previous study in the case of dyes biosorption [47].

To our knowledge, the use of biosorbents based on microbial biomass immobilized on natural polymers have not been previously investigated for the removal of CPX from water matrix.

Considering the negative impact of CPX on the environment and the use of the biosorption process as a removal method, this paper focused on the synthesis, characterization and biosorption capacity evaluation of *Saccharomyces cerevisiae* immobilized in calcium alginate beads as a biosorbent to remove CPX from aqueous solutions.

## 2. Materials and Methods

### 2.1. Chemicals and Analytical Procedure

All the chemicals (cephalexin (Cayman Chemical, Tallinn, Estonia), sodium hydroxide (Chempur, Piekary Śląskie, Poland), hydrochloride acid (Chemical Company, Iași, Romania), sodium alginate (Carl Roth, Karlsruhe, Germany), calcium chloride (Chempur, Piekary Śląskie, Poland), sodium chloride (Chemical Company, Iași, Romania), ethanol (Chemical Company, Iași, Romania)) required in the experiments were of analytical purity and were used without further purification. 

Yeast strain of *Saccharomyces cerevisiae* was kindly donated by Rompak Company (Pașcani, Romania).

**Figure 1:**
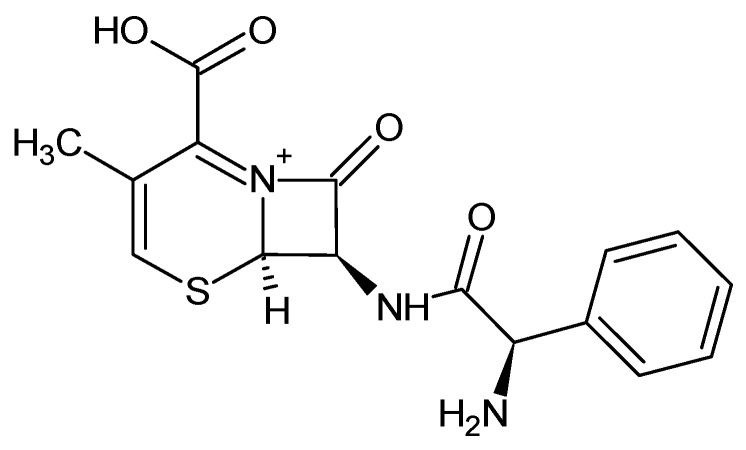
Chemical structure of cephalexin. (CAS 15686-71-2; molecular formula: C_16_H_17_N_3_O_4_S; MW = 347.40 g/mol).

A stock solution of cephalexin (Figure 1) with a concentration of 500 mg/L was prepared by dissolving the reagent in distilled water and kept at 4 °C in a closed vessel. 

For the calibration curve, 0.02, 0.1, 0.2, 0.3, 0.4, 0.5 and 0.6 mL of CPX stock solution were placed in a series of volumetric flasks and the volumes were adjusted to 10 mL in order to obtain concentrations ranging between 1 mg/L and 30 mg/L. The samples absorbance was acquired at 260 nm with the help of a UV1280 spectrophotometer (Shimadzu, Tokyo, Japan). A calibration graph (absorbance vs. concentration) was plotted and a linear regression equation was recovered. 

The other work solutions were obtained by appropriate dilutions. When necessary, NaOH (0.1 M) or HCl (0.1 M) were used to adjust the pH.

### 2.2. Biosorbent Preparation 

**Figure 2:**
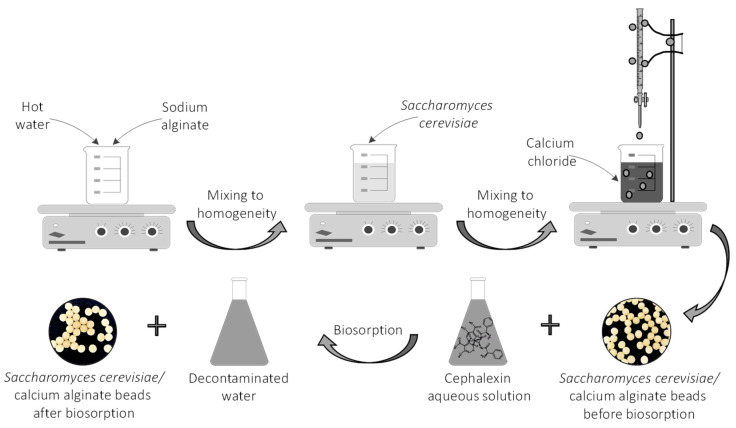
Schematic representation of the CPX biosorption process.

A certain amount of sodium alginate (for a 1% concentration) was introduced in a laboratory beaker containing hot water (70 °C) (Figure 2) and mixed on a Nahita magnetic stirrer (Auxilab, Beriain, Spain) until complete dissolution. Inactivated dried *Saccharomyces cerevisiae* yeast was added in order to attain a final concentration of 2.5% d.w. The mixing process was continued until a homogeneous suspension was obtained. The product was placed in a burette and dripped in a 2% calcium chloride solution. 

The resulting *Saccharomyces cerevisiae*/calcium alginate beads were separated, washed and then stored in calcium chloride solution for 24 h at 4 °C. In all the biosorption experiments, they were used in their wet form.

### 2.3. Biosorbent Characterization (Morphology, Functional Groups, Point of Zero Charge)

The surface morphology and elemental composition of the synthesized *Saccharomyces cerevisiae/*calcium alginate beads were examined before and after the biosorption process by using a scanning electron microscope (SEM Quanta 200 3D (FEI Europe B.V., Eindhoven, Netherlands)) equipped with an energy-dispersive X-ray system. The dried beads (50 °C, 2 h in an Air Performance AP60 hot air oven, (Froilabo, Paris, France) were fixed to stubs with double adhesive carbon discs. The analyses were carried out in normal secondary electron mode (SE) in low vacuum. The detection was ensured by a large field detector (LFD) at an accelerating voltage of 20 kV, a working distance of 15 mm and a spot size of 5. The magnification range was between 1 mm and 10 µm. 

The Fourier transform infrared (FT-IR) allowed the identification of the functional groups existing in the biosorbent material. Spectra were recorded with the attenuate total reflection (ATR) detection method before and after the biosorption process from 4000 to 400 cm^−1^ (32 scans co-added) with a resolution of 4 cm^−1^ on a Nicolet iS50 FT-IR spectrometer (Thermo Scientific, Dreiech, Germany) coupled with a built-in ATR accessory, DTGS detector and a KBr beam splitter. The ATR plate was cleaned with ethanol after each spectrum acquisition. Air was used for background spectrum reference, which was registered and compared with the anterior one. 

The biosorbent point of zero charge was established by pH drift method [34,48]. Aliquots of 25 mL of a 0.1 M NaCl solution were used as background electrolyte. The initial pH values (pH_i_) were adjusted between 2 and 12 by small additions of HCl (0.1 M) or NaOH (0.1 M) and measured with a portable pH meter (Dostmann KLH9.1, 0–14 pH, Carl Roth, Karlsruhe, Germany), and 0.5 g of biosorbent were added to each solution. After 24 h of magnetically stirring at room temperature, the final pH values (pH_f_) were measured again. The pH_pzc_ of the sample was determined from the curve pH_f_ = f(pH_i_).

### 2.4. Effect of Biosorption Parameters (pH, Biosorbent Amount, CPX Initial Concentration)

CPX (50 mg/L) solutions with different pH (2–12) were put in contact with 1 g of biosorbent beads. Once the favorable pH value was established, the amount of the biosorbent was varied (0.5–3 g). After that, CPX concentration was changed between 10 mg/L and 80 mg/L. For these determinations, the contact period was set at 12 h. 

Each time, volumes of 25 mL of CPX solution were used. The remaining CPX concentrations were calculated by reading the samples absorbance at the wavelength of 260 nm against the calibration curve.

The CPX removal efficiency (*R*, %) and the biosorption capacity at equilibrium (*q_e_,* mg/g) were determined with the following equations:

(1) $$R=\frac{(C_{0}-C_{e})\cdot 100}{C_{0}}$$

(2) $$q_{e}=\frac{\left(C_{0}-C_{e}\right)\cdot V}{m}$$

where *C_0_* and *C_e_* are the initial and at equilibrium state concentrations (mg/L); *V* is the CPX volume (L) and *m* is the amount of the biosorbent (g).

### 2.5. Kinetics and Equilibrium Isotherms

Two kinetic models, namely pseudo–first-order (Equation (3)) and pseudo–second-order (Equation (4)) were tested.

(3) $$\log(q_{e}-q_{t})=\log qe-\frac{k_{1}}{2.303}\cdot t$$

(4) $$\frac{t}{q_{t}}=\frac{1}{k_{2}\cdot q_{e}^{2}}+\frac{t}{q_{e}}$$

where *q_t_* and *q_e_* are the biosorption capacities of CPX at time *t* and at equilibrium, mg/g; *t* is the reaction time, minutes; *k_1_* and *k_2_* are the biosorption rate constants for pseudo–first-order and pseudo–second-order kinetic models, respectively, min^−1^.

In terms of equilibrium isotherms, Langmuir (Equation (5)) and Freundlich (Equation (6)) ones were considered to investigate the CPX biosorption on the prepared biosorbent.

(5) $$\frac{C_{e}}{q_{e}}=\frac{1}{q_{m}\cdot K_{L}}+\frac{C_{e}}{q_{m}}$$

(6) $$\log q_{e}=\log K_{F}+\frac{1}{n}\cdot \log C_{e}$$

where *C_e_* and *q_e_* are the equilibrium concentration (mg/L) and equilibrium biosorption capacity (mg/g); *q_m_* is the maximum biosorption capacity (mg/g); *K_L_* is the Langmuir constant (L/g); *n* and *K_F_* (mg/g) are the Freundlich constants. 

## 3. Results and Discussion

### 3.1. Biosorbent Preparation

**Figure 3:**
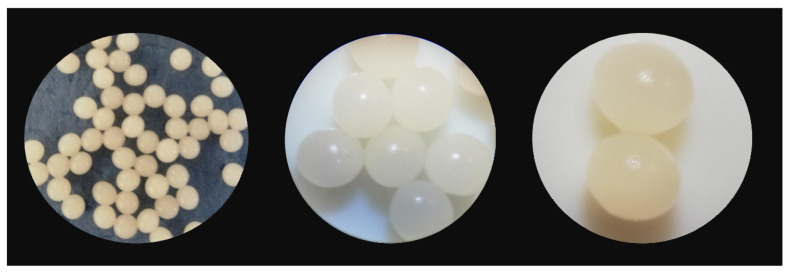
*Saccharomyces cerevisiae*/calcium alginate beads.

For the biosorbent preparation, a homogeneous solution of sodium alginate in hot water was firstly obtained. The appropriate amount of *Saccharomyces cerevisiae* biomass was added and mixed for several hours. The suspension was then dropped from a burette into a calcium chloride solution. The biosorbent shape (Figure 3) depends on the alginate solution viscosity, calcium chloride collection solution concentration and temperature, and on the stirring speed. Spherical beads with a mean diameter of 3.32 ± 0.12 mm and opaque, shiny appearance were obtained. Their aspect was stable even after one week of storage at 4 °C. 

### 3.2. SEM and FT-IR Analyses of the Biosorbent 

**Figure 4:**
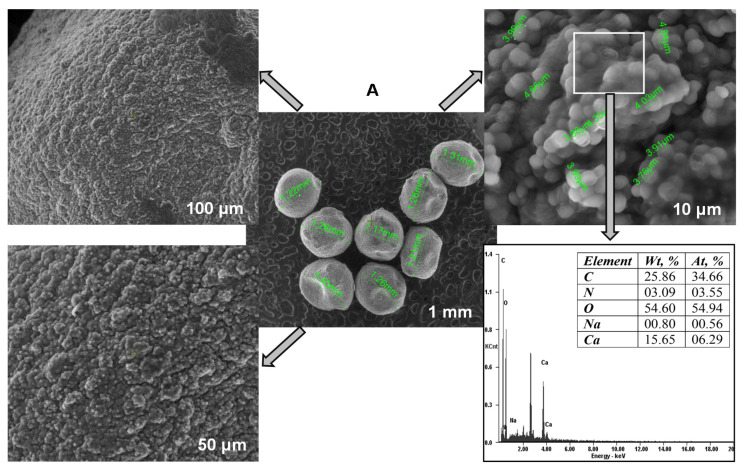

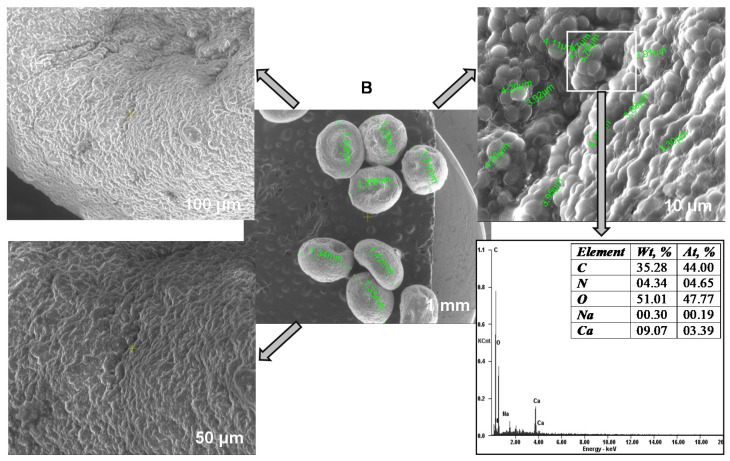
SEM images and EDAX analyses of the biosorbent before (**A**) and after biosorption (**B**).

Scanning electron microscopy served to study the *Saccharomyces cerevisiae*/calcium alginate beads morphology (Figure 4A). The internal mesoporous structure can be easily observed. SEM pictures show that the microorganism was homogeneously distributed in the polymer matrix, confirming that the immobilization process successfully took place. When analyzing the SEM photographs presented in Figure 4B, some differences can be noted, among them being the fact that the particles were smoother after CPX biosorption. The biosorbent elemental analysis revealed higher percentages of carbon and nitrogen at the end fact that sustains the antibiotic removal by biosorption on the material surface. The difference between the amount of calcium before and after the biosorption can be explained by the fact that most of the calcium ions were removed in the washing preliminary step required before the biosorbent-adsorbate contact.

**Figure 5:**
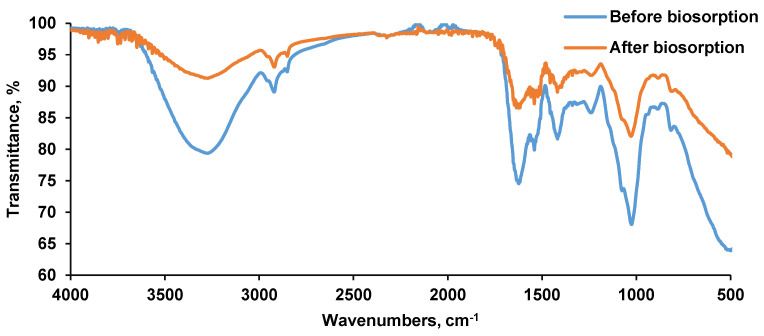
FT-IR spectra of the biosorbent before and after biosorption.

Figure 5 depicts the *Saccharomyces cerevisiae*/calcium alginate beads FT-IR spectra. Before the biosorption, the vibrations characteristics ranged between 3200 and 2900 cm^−1^ and between 1600 and 400 cm^−1^. The broad peak recorded at 3273 cm^−1^ was specific to the OH group while the sharp one of 2920 cm^−1^ can be assigned to C–H stretching. Signals of 1623, 1540, 1418, 1239 cm^−1^ and 1025 cm^−1^ can be attributed to C=O, amide II (N–H join with C–N), asymmetric COO^−^ and C–O stretching, respectively [49,50]. The peak registered at 815 cm^−1^ was specific to carbohydrates C–H vibration. At 1646 cm^−1^ a vibration of amide was recorded; at 1635 cm^−1^ a peak of carbonyl group C=O can be remarked. At 1362 cm^−1^, at 1313 cm^−1^ and at 1238 cm^−1^, stretching for C-OH, for amide III (proteins) and for PO_2_–asymmetric and symmetric (phosphorylated proteins, phospholipids) can be distinguished [51,52]. 

After the biosorption, some of the above mentioned peaks gained in intensity. As an example, the signal of 3273 cm^−1^ specific for hydroxyl group gained in intensity from 79.352 to 91.246%; the signal of 2920 cm^−1^ specific for C–H band gained in intensity from 89.095 to 93.039%; the signal of 1623 cm^−1^ specific for C=O stretching gained in intensity from 74.535% to 86.547%; the signal of 1540 cm^−1^ specific for amidic group gained in intensity from 79.877% to 87.230% and the signal of 1418 cm^−1^ specific for carboxylic group gained in intensity from 81.642% to 89.085%. These results reveal that the CPX was retained on the biosorbent material. 

### 3.3. Biosorbent Point of Zero Charge (pH_PZC_)

**Figure 6:**
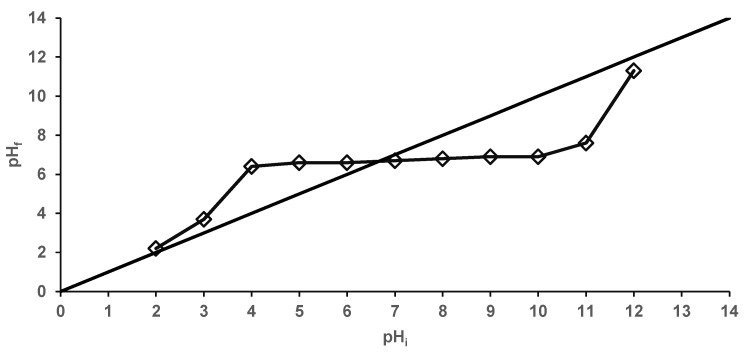
Biosorbent point of zero charge.

At given work conditions, the point of zero charge (also called the isoelectric point) is the pH value at which there are equal amount of positive and negative charges on the biosorbent surface [53]. When the pH is lower than the pH_PZC_, the biosorbent is considered positively charged. On the contrary, when pH is above pH_PZC_, the biosorbent is in negative charge. Figure 6 illustrates the difference between the initial and final pH values. 

It can be seen that the pH_PZC_ value of the prepared material was 6.6, indicating a rather neutral character. This observation is consistent with the one published by de Rossi et al. [48], which reported that beads obtained from residual *Saccharomyces cerevisiae* biomass immobilized on calcium alginate had a pH_PZC_ of 7.0.

### 3.4. Effect of pH

**Figure 7:**
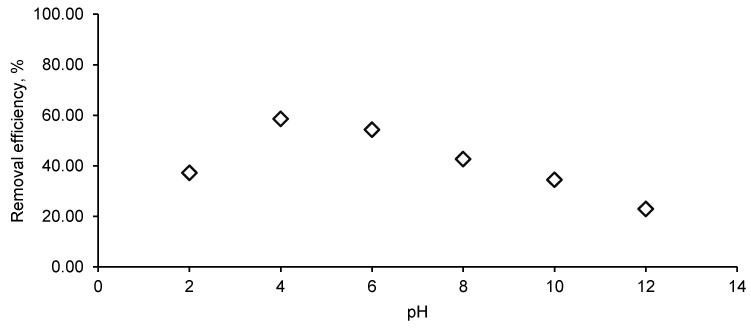
pH influence on CPX removal efficiency. (CPX solution volume: 25 mL; CPX initial concentration: 50 mg/L; biosorbent dose: 1 g/L).

One of the main factors affecting the biosorption process is represented by the initial pH value of the working solution (Figure 7). 

In our case, the CPX removal efficiency had the highest value in acidic media (58.56%) at pH 4 and the lowest one in the alkaline environment (22.92% at pH 12). In correlation with pH_PZC_, pH offers information about the biosorption process. At a pH inferior to pH_PZC_, the biosorbent possesses a positive charge and can interact with the negative charges of the antibiotic, while at a pH higher than pH_PZC,_ interactions take place between the negative biosorbent surface and the medicine positive charges. 

At the same time, the functional groups existing in CPX structure lead to the existence of two pKa values: pKa_1_ = 2.56 and pKa_2_ = 6.88. Below pKa_1_, CPX is in its cationic form, between pKa_1_ and pKa_2_ it is in zwitterionic form and after pKa_2_ in its anionic form. Therefore, the high value recorded at pH 4 for the biosorption is explained by the electrostatic attraction between the biosorbent positive surface and the CPX zwitterionic form. Similar details have been described by other researchers [29,35,39,54] who studied the CPX biosorption alone or in combination with other drugs on different types of biosorbent materials. They also reported that at a strong acid or alkaline pH, the biosorption capacity is lower due to competition between the hydrogen ions or hydroxyl groups, respectively with CPX ions for the biosorbent binding sites.

### 3.5. Effect of Biosorbent Dosage

**Figure 8:**
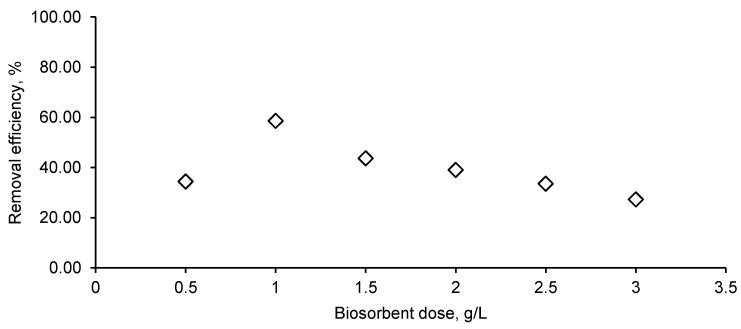
Biosorbent dose influence on CPX removal efficiency (CPX solution volume: 25 mL; CPX initial concentration: 50 mg/L; CPX initial solution pH: 4).

Various biosorbent amounts (0.5, 1, 1.5, 2, 2.5, 3 g/L) were added to 25 mL of CPX solution with a concentration of 50 mg/L at pH 4. As shown in Figure 8, the removal efficiency increased from 34.43% for a biosorbent dose of 0.5 g/L to more than 50% when the biosorbent concentration was of 1 g/L, and began to decrease when the biosorbent was used in higher quantities. 

Comparable observations were drawn by Samarghandi et al. [39], who focused on the removal of CPX from aqueous solution by using zeolites. They identified that the increase in the biosorbent quantity ensures a larger surface area for biosorption and that after a certain biosorbent dose, aggregation of available binding sites appears, leading to a lower removal efficiency. Bangari et al. [35] presented a similar interpretation for the influence of the biosorbent material dosage on the biosorption of three different antibiotics, CPX being one of them. As a consequence, for further experiments, a biosorbent concentration of 1 g/L was considered as optimum. 

### 3.6. Effect of CPX Initial Concentration

**Figure 9:**
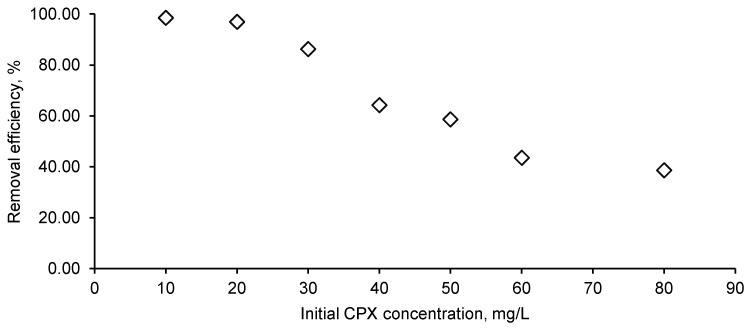
Initial CPX influence on the removal efficiency (CPX solution volume: 25 mL; CPX initial solution pH: 4; biosorbent dose: 1 g/L).

Volumes of 25 mL of CPX solution with concentrations between 10 mg/L and 60 mg/L with pH adjusted at four were put in contact with 1 g/L of biosorbent for 12 h. Figure 9 displays the influence of CPX initial concentration on the biosorption process. At low values (10 mg/L and 20 mg/L), the antibiotic was almost completely removed while an augmentation of CPX concentration at 80 mg/L led to a removal efficiency of only 38.61%. The same behavior was disclosed by Nazari et al. [16,55], who conducted their studies of CPX biosorption on activated carbon obtained from walnut shell and showed that the retention is more favorable at a reduced concentration. 

### 3.7. Biosorption Kinetics

For the kinetic study, 1 g/L biosorbent was introduced in a flask containing 50 mL of CPX aqueous solution with a concentration of 30 mg/L at pH set at four. Every 5 to 10 min samples were collected and their antibiotic amount determined.

**Figure 10:**
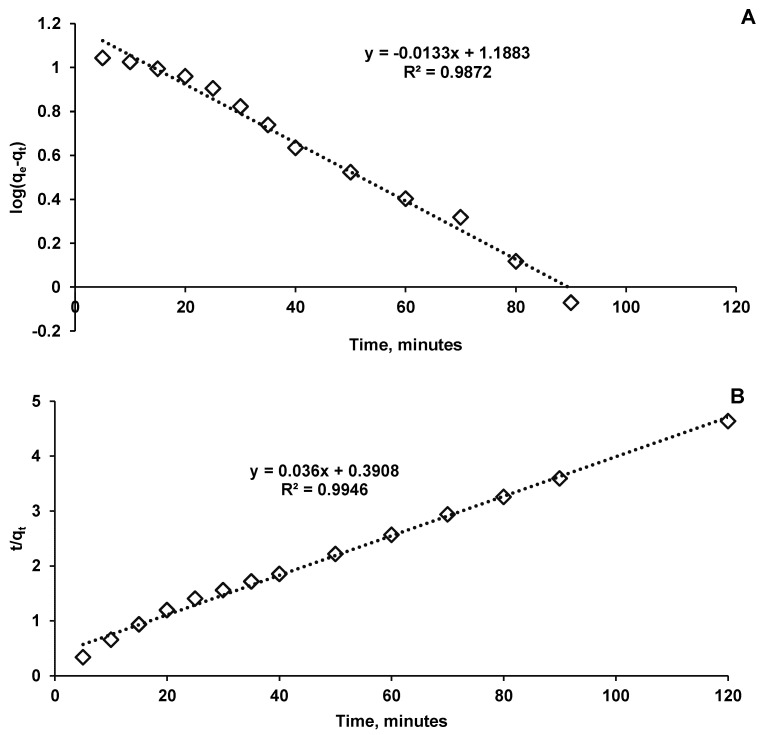
Pseudo–first-order (**A**) and pseudo–second-order (**B**) kinetics models of CPX biosorption process.

In order to analyze the rate controlling step in the elimination of CPX, two kinetic models, the pseudo–first-order and pseudo–second-order were explored (Figure 10).

Biosorption kinetic parameters of CPX on *Saccharomyces cerevisiae*/calcium alginate beads were calculated using the equations presented in Figure 10 (Table 1). The correlation coefficients values revealed that the pseudo–second-order model better fit the experimental data than the pseudo-first order one. In the last situation, even though until 90 min R^2^ is 0.9872 when the model is extended, for all the 120 min considered, a decrease in the correlation coefficient can be detected. Taking into account the kinetic model, it can be considered that chemical interaction between the biosorbent and the antibiotic represents one of the explanations of the biosorption process mechanism, which is also in agreement with the findings of Zhao et al. [41], who sustained the chemisorption process of CPX on a synthetic biosorbent material.

### 3.8. Biosorption Isotherms

The biosorption can be described as a process in which the adsorbate is transferred on the biosorbent material. The retention mechanisms consist of chemical and physical adsorption represented by formation of chemical bonds and by interactions such as Van der Waals and ion exchange, respectively [56], and are the subject of different modelling investigations. Langmuir and Freundlich models are known as the most used for study of the experimental data at equilibrium. 

**Figure 11:**
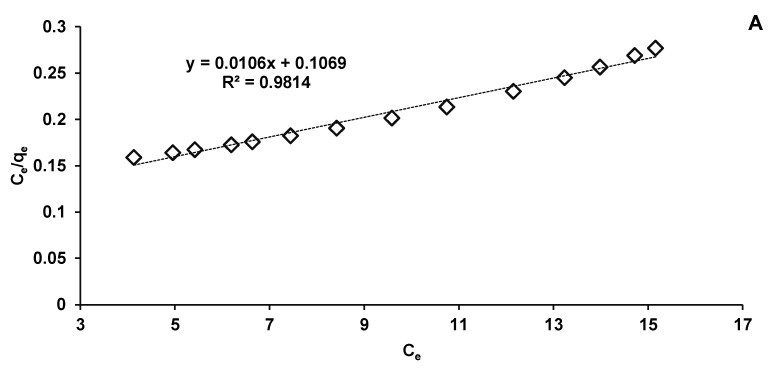

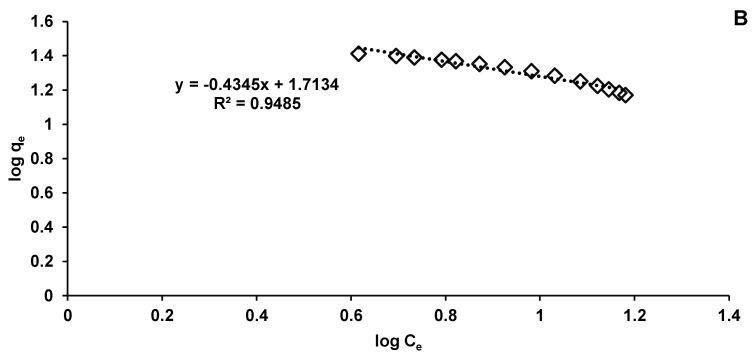
Langmuir (**A**) and Freundlich (**B**) isotherms of CPX biosorption process.

In our research, the collected data fit better on the Langmuir model (R^2^ = 0.9814) (Figure 11A) than on the Freundlich one (R^2^ = 0.9485) (Figure 11B). These biosorption isotherms indicated the interaction of the adsorbate with the tested biosorbent providing valuable information on its biosorption capacity. Langmuir isotherm is based on hypotheses such as monolayer biosorption, homogeneity of biosorption sites distribution, constancy of biosorption energy and insignificant interactions between the molecules to be adsorbed. Freundlich isotherm stipulates that the biosorption phenomenon is a multilayer one and that it occurs on heterogeneous surfaces. 

Table 2 presents the biosorption parameters released from the Langmuir and Freundlich equations according to Figure 11. 

For the first mentioned isotherm model, the maximum adsorption capacity of CPX on *Saccharomyces cerevisiae*/calcium alginate beads was of 94.34 mg/g, significantly lower than that calculated by Bangari et al. [35], who conducted absorption of CPX on boron nitride nanosheets, but higher than that indicated by Acelas et al. [57], who used palm oil fiber as a precursor for an adsorbent material able to remove CPX from aqueous solutions. 

Immobilization of microorganisms creates a better contact between the biosorbent and CPX molecules. As presented in Table 3, the values obtained for the maximum biosorption capacity predicted by the Langmuir model were higher than those reported in the literature for living and non-living microorganism/biomass in their free state. 

The Langmuir model also serves to establish the value of the separation factor (R_L_) which reveals if the biosorption is irreversible (R_L_ = 0), linear (R_L_ = 1), favorable (0 < R_L_ < 1), or unfavorable (R_L_ > 1) [34]. Our calculated R_L_ is 0.7104 indicating a favorable CPX elimination process. 

In the case of the Freundlich model, k_F_ and n constants give details on the amount of CPX adsorbed on biosorbent surface and on the biosorption intensity. The k_F_ value suggests a favorable biosorption. This fact is in a good agreement with the results of Yadav et al. [58], who synthesized a new polymeric material and used it to eliminate different dyes, drugs and metals from the water matrix. 

## 4. Conclusions

The biosorbent *Saccharomyces cerevisiae* immobilized in calcium alginate composite beads was successfully synthesized. It had a mesoporous structure and the microorganism was homogeneously distributed in the polymer matrix, confirming that the immobilization process took place. This aspect is confirmed by results of SEM and FTIR analysis.

The best biosorption efficiency for CPX was obtained at an acidic pH (four), a fact explained by the biosorbent mesoporous structure, the pH_PZC_ value of 6.6 and the zwitterionic form of CPX at this pH value.

Biosorption studies of CPX on *Saccharomyces cerevisiae* immobilized in calcium alginate beads indicate that the pseudo–second-order kinetic model is best suited to describe the process of kinetics.

The isotherms analysis evidenced that the CPX biosorbent interaction has a good fit for the Langmuir model with a correlation coefficient of 0.9814 and a maximum biosorption capacity of 94.34 mg/g.

The findings of this study indicate that immobilization technique leads to obtain the biosorbents based on microorganisms which are easy to handle, stable, low-cost, eco-friendly and have the potential to be applied in the treatment of drugs-contaminated water or wastewater treatment plant effluents.

## Figures and Tables

**Table 1:** Biosorption kinetic parameters of CPX on *Saccharomyces cerevisiae*/calcium alginate beads.

Parameter	Pseudo–First-Order Kinetic Model	Pseudo–Second-Order Kinetic Model
q_e_ (mg/g)	15.4276	25.89
k_1_ (min^−1^)	0.0306	-
k_2_ (min^−1^)	-	0.2529
R^2^	0.9872	0.9946

**Table 2:** Langmuir and Freundlich models of CPX biosorption process on *Saccharomyces cerevisiae*/calcium alginate beads.

Parameter	Langmuir Model	Freundlich Model
Q_m_ (mg/g)	94.34	-
k_L_ (L/mg)	0.9915	-
R_L_	0.7104	-
k_F_ (mg/g)	-	5.5428
n_F_	-	2.3068
R^2^	0.9814	0.9485

**Table 3:** Biosorption capacities of various biosorbents investigated for the removal of CPX from aqueous solutions.

Biosorbent	Maximum Biosorption Capacity, Q_m_ [mg/g]	References
*Chlorella* sp., biomass (non-living)	63.29	[44]
Bacterial consortium biomass *Burkholderia cepacia, Chryseomonas luteola, Pseudomonas fluorescens, Bacillus subtilis, Bacillus megaterium, Bacillus stearothermophilus, Citrobacter freundii, Kluyvera* spp. (living)	10.61	[43]
Mixed bacterial cell biomass (Bacteria genus: *Bacillus*, *Pseudomonas*, *Burkholderia*, *Chryseomonas*, *Citrobacter*, *Klyuvera*)		[45]
- living - non-living	60 30	
Mixed Gram-positive bacteria (*Bacillus* genus)		
- living - non-living	50.91 15.99	
Mixed Gram-negative bacteria (bacteria genus: *Pseudomonas*, *Burkholderia*, *Chryseomonas*, *Citrobacter*, *Klyuvera*)		
- living - non-living	40.44 25.11	
*Bacillus subtilis* strain (living)	27.22	[46]
*Saccaromyces cerevisiae* immobilized in calcium alginate beads	94.34	Present study

## Data Availability

All data produced in this study are presented in this paper.
